# A novel method for transforming the thermophilic bacterium *Geobacillus kaustophilus*

**DOI:** 10.1186/s12934-018-0969-9

**Published:** 2018-08-17

**Authors:** Megumi Miyano, Kosei Tanaka, Shu Ishikawa, Kotaro Mori, Andrés Miguel-Arribas, Wilfried J. J. Meijer, Ken-ichi Yoshida

**Affiliations:** 1 Department of Science, Technology and Innovation, Kobe University, 1-1 Rokkodai, Nada, Kobe, 657 8501 Japan; 2 Centro de Biología Molecular ‘Severo Ochoa’ (CSIC-UAM), Instituto de Biología Molecular ‘Eladio Viñuela’ (CSIC), Universidad Autónoma, Canto Blanco, 28049 Madrid, Spain

**Keywords:** *Geobacillus kaustophilus*, *Bacillus subtilis*, Plasmid, Conjugation, Mobilization, Transformation

## Abstract

**Background:**

Bacterial strains of the genus *Geobacillus* grow at high temperatures of 50–75 °C and could thus be useful for biotechnological applications. However, genetic manipulation of these species is difficult because the current techniques for transforming *Geobacillus* species are not efficient. In this study, we developed an easy and efficient method for transforming *Geobacillus kaustophilus* using the conjugative plasmid pLS20cat.

**Results:**

We constructed a transformation system comprising (i) a mobilizable *Bacillus subtilis*–*G. kaustophilus* shuttle plasmid named pGK1 that carries the elements for selection and replication in *Geobacillus*, and (ii) a pLS20cat-harboring *B. subtilis* donor strain expressing the *dam* methylase gene of *Escherichia coli* and the conjugation-stimulating *rap*_LS20_ gene of pLS20cat. This system can be used to efficiently introduce pGK1 into *G. kaustophilus* by mobilization in a pLS20cat-dependent way. Whereas the thermostable kanamycin marker and *Geobacillus* replication origin of pGK1 as well as expression of *dam* methylase in the donor were indispensable for mobilization, ectopic expression of *rap*_LS20_ increased its efficiency. In addition, the conditions of the recipient influenced mobilization efficiency: the highest mobilization efficiencies were obtained using recipient cells that were in the exponential growth phase. Furthermore, elimination of the origin of transfer from pLS20cat enhanced the mobilization.

**Conclusions:**

We describe a novel method of plasmid mobilization into *G. kaustophilus* recipient from *B. subtilis* donor depending on the helper function of pLS20cat, which enables simple, rapid, and easy transformation of the thermophilic Gram-positive bacterium.

## Background

**Fig. 1:**
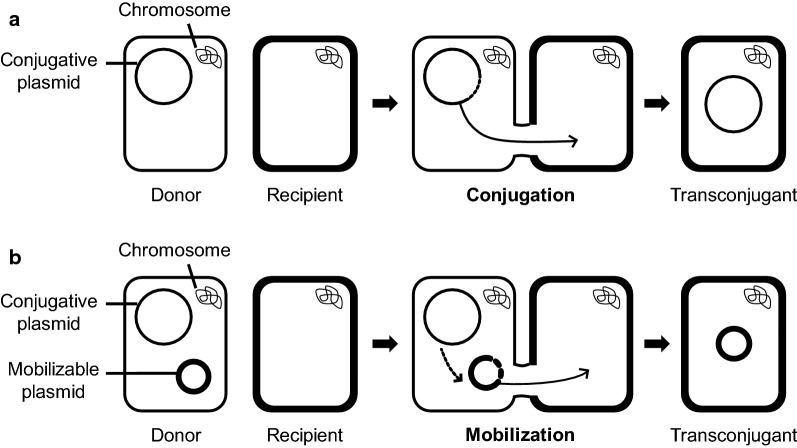
Schematic presentation of conjugation and mobilization. Conjugation refers to transfer of a conjugative plasmid from donor to recipient (**a**), whereas mobilization refers to mobilization of a mobilizable plasmid from donor to recipient mediated by the helper function of a co-resident conjugative plasmid (**b**). Large light circle, conjugative plasmid; small bold circle, mobilizable plasmid; light rounded rectangle, donor cell; and bold rounded rectangle, recipient cell

In general, bacteria reproduce asexually and their genetic traits are inherited vertically from mother to daughter cells. However, they can also acquire different traits from other species via horizontal gene transfer (HGT), a mechanism that contributes importantly to the genetic diversity of bacteria [1–4]. Three different mechanisms are mainly responsible for HGT: transformation, transduction, and conjugation. Transformation involves the acquisition of naked DNA from the extracellular environment [5], transduction involves transfer of genetic information through bacteriophage infection [6], and conjugation involves physical cell-to-cell contact for DNA transfer, mediated by conjugative elements that can be embedded in the bacterial genome (named Integrative Conjugative Element, ICE) or present on plasmids (named conjugative plasmids) [7]. A conjugative element renders a complete set of genes required for DNA transfer. A cell harboring a conjugative plasmid can act as a donor to transfer the plasmid to a recipient cell lacking the plasmid. Conjugation has important environmental and medical implications. In addition, conjugation is exploited as a tool for genetic modification of bacteria thereby serving research and industrial purposes. Plasmids can be classified into two categories: conjugative and non-conjugative; of which only the former can transfer themselves to recipient cells (Fig. 1; in most cases, only one DNA strand of the conjugative plasmid is transferred into the recipient cell through a pore that connects the donor and recipient cells). One of the initial steps of the conjugation route involves so-called relaxosome proteins processing the DNA to generate the single-stranded DNA, which is transferred to the recipient cell. The key enzyme of the relaxosome complex is a relaxase that recognizes and binds to specific sequences in a region on the plasmid named the origin of transfer (*oriT*). After binding, the relaxase cleaves the DNA in a strand- and site-specific manner at a specific position, named the *nic* site, and remains covalently attached to the 5′-end of the nicked strand. Elongation of the DNA at the generated hydroxyl group at the 3′-end of the *nic* site causes displacement of the strand at which the relaxase is attached. The relaxase and its attached DNA is delivered to the pore and subsequently transferred into the recipient cell. As may be expected, many plasmids lacking the conjugation genes cannot be transferred. However, despite lacking the conjugation genes, a rather large group of plasmids can be transferred when they are co-resident with a conjugative plasmid via a process named mobilization (Fig. 1). Mobilizable plasmids can be divided into two groups. Members of both groups transfer their ssDNA strand through the connecting pore generated by the conjugative plasmid. Plasmids belonging to one of these groups encode their proper relaxase that acts on the cognate *oriT* present on the plasmid; the relaxase and *oriT* of the plasmids are unrelated to those present on the conjugative plasmid. Plasmids of the other group do not encode a relaxase gene; they merely contain a copy of the *oriT* that is present on the conjugative plasmid.

The genus *Geobacillus* was first described in 2001 [8]. It comprises thermophilic bacteria that were previously included in the genus *Bacillus*. Geobacilli are Gram-positive, endospore-forming, aerobic or facultative anaerobic thermophiles and are isolated from various environments, such as soil, hot springs, oilfields, and hay compost [9–12]. Geobacilli grow optimally at 50–75 °C. This feature makes them beneficial for biotechnological applications. For instance, the high temperature prevents the growth of possible contaminant mesophilic bacteria, and also saves energy and costs to remove fermentation heat. In addition, it can facilitate the recovery of volatile products from culture media. Successful use of bacterial species for biotechnological applications requires efficient ways to modify it genetics in order to engineer strains with desired and/or optimized features. A few techniques to modify *Geobacillus* species are available [13]. For example, a protoplast method for *Geobacillus stearothermophilus* NUB36 [14] and an electroporation method for *Geobacillus thermoglucosidasius* DL44 [15] have been reported. However, besides that these techniques are not efficient, transformation by the protoplast technique is laborious and the electroporation method requires the optimized conditions for each strain. Here, we describe a novel method to modify *Geobacillus kaustophilus* strain HTA426 that is based on interspecies mobilization. The technique is simple, rapid and reproducible.

*Geobacillus kaustophilus* HTA426 can grow in lysogeny broth (LB) medium at high temperatures ranging from 42 to 74 °C under aerobic conditions as rapidly as *Escherichia coli* at 37 °C. Growth has low nutrient demands and various carbon sources can be used, which include glycerol, casamino acids, hexoses (d-glucose, d-galactose, d-mannose, and *myo*-inositol), pentoses (l-arabinose and d-xylose), oligosaccharides (cellobiose, maltose, sucrose, soluble starch, and xylooligosaccharide), and alcohols (ethanol, 2-propanol, and *n*-butanol) [16]. Previously, a technique for the genetic manipulation of *G. kaustophilus* HTA426 was described that is based on the transfer of mobilizable plasmids from *E. coli* to HTA426 mediated by the broad host-range conjugative plasmid pUB307 [16]. This technique involved an elaborate device to overcome the redundant restriction-modification (RM) systems. Three restriction modification (RM) systems are reported to be functional in HTA426: system one composed of genes *GK0343* (M subunit), *GK0344* (S subunit) and *GK0346* (R subunit); system two composed of genes *GK1380* (M subunit), *GK1381* (S subunit) and *GK1382* (R subunit); and system three comprised by genes *GKP09* (endonuclease) and *GKP08* (methylase). GKP08 methylates a sequence that is very similar to the one recognized by the *E. coli dam* methylase, which is responsible for a methylation pattern similar to that of *E. coli dam* (5′-G^*N*6m^ATC-3′), and the use of an *E. coli* donor strain that is *dam*^+^ functionally compensates for the GKP08 methylase [16]. However, the methylases of the other two RM systems needed to be expressed in the *E. coli* donor cell to properly methylate the corresponding recognition sites. To circumvent this inconvenience, a derivative of HTA426 was constructed and named MK244, that lacks the RM systems one and two [17]. The use of this engineered recipient strain alleviated the use of the special *E. coli* expressing the corresponding methylases. Besides the transfer of mobilizable plasmids into *G. kaustophilus*, a modification of this method was also developed to manipulate regions of the bacterial genome involving a counter-selection system [18]. Nevertheless, despite these advances the transformation efficiencies obtained with this method were low and not always successful in the case of the counter-selective system. Moreover, the method was very laborious and time-consuming, requiring at least 6 h of incubation on solid media for mating.

pLS20, a conjugative plasmid isolated from *B. subtilis* natto [19], can transfer itself to various *B. subtilis*-related Gram-positive bacteria, including *Bacillus anthracis*, *Bacillus cereus*, *Bacillus licheniformis*, *Bacillus megaterium*, *Bacillus pumilus*, and *Bacillus thuringiensis* [20]. pLS20cat, a derivative of pLS20 carrying a chloramphenicol resistance gene possesses the outstanding ability of rapid transfer: after simply mixing liquid cultures of donor and recipient cells the plasmid is efficiently transferred within 15 min [21–26]. In addition, pLS20cat can mobilize a co-resident plasmid if it contains a functional copy of the *oriT* of pLS20cat (*oriT*_LS20_) [26, 27]. Plasmid pLS20 or a derivative has been used to mobilize plasmids in various studies [20, 28–30]. Moreover, it was recently used to mobilize a large chromosomal segment [31].

In this study, we exploited the features of pLS20cat to develop a versatile, rapid, and easy transformation system for *G. kaustophilus*. We show that pLS20cat enabled the mobilization of a plasmid into *G. kaustophilus*. Efficiency of mobilization could be enhanced in three ways; (i) by ectopically expressing the pLS20cat *rap*_LS20_ gene, which encodes the anti-repressor of the conjugation operon, in the donor cells, (ii) by using exponentially growing recipient cells, and (iii) by elimination of *oriT*_LS20_ on pLS20cat.

## Methods

### Bacterial strains and growth conditions

Bacterial strains and plasmids used in this study are listed in Table 1. Synthetic oligonucleotides used as PCR primers are shown in Table 2. Bacterial strains were grown on LB medium (Difco). When needed, the medium was supplemented with antibiotics: 5-mg L^−1^ chloramphenicol, 1-mg L^−1^ erythromycin, 100-mg L^−1^ spectinomycin, 8-mg L^−1^ phleomycin, or 10-mg L^−1^ kanamycin.

**Table 1:** Strains and plasmids used in this study

Strain and plasmid	Description	Source or references
Strain		
*B. subtili*s		
GR138	*trpC2* pLS20cat pGR16B	[27]
GR23	*trpC2 amyE*::(P*spank*–*rap*_LS20_ *spc*) pLS20cat	[27]
PKS11	*trpC2* pLS20cat	[22]
PKS86	*trpC2 amyE*::(P*spank*–*rco*_LS20_ *spc*) pLS20rco	[23]
STM1	*trpC2 epr*::(P*rpsO*–*dam ble*) pLS20cat	This study
TSU077	*trpC2 epr*::(P*rpsO*–*dam ble*)	This study
YNB051	*trpC2 amyE*::(P*spank*–*rap*_LS20_ *spc*) *epr*::(P*rpsO*–*dam ble*) pLS20cat pGK1	This study
YNB052	*trpC2 amyE*::(P*spank*–*rap*_LS20_ *spc*) *epr*::(P*rpsO*–*dam ble*) pLS20cat pGK2	This study
YNB059	*trpC2 amyE*::(P*spank*–*rap*_LS20_ *spc*) *epr*::(P*rpsO*–*dam ble*) pLS20cat	This study
YNB042	*trpC2 amyE*::(P*spank*–*rap*_LS20_ *spc*) pLS20cat pGK1	This study
YNB032	*trpC2 epr*::(P*rpsO*–*dam ble*) pLS20cat pGK1	This study
YNB101	*trpC2 amyE*::(P*spank*–*rap*_LS20_ *spc*) *epr*::(P*rpsO*–*dam ble*) pLS20catΔ*oriT* pGK1	This study
*G. kaustophilus*		
MK244	Δ*pyrFR* Δ*GK1378*–*GK1390* Δ*GK0343*–*GK0346*	[17]
MK72	Δ*pyrFR*	[18]
Plasmid		
pGK1	pGR16B containing the replication origin of pUCG18T and *kan* (thermostable)	This study
pGK2	pGR16B containing *kan* (thermostable)	This study
pGR16B	*erm kan* (thermostable) *oriT*_LS20_	[27]
pLS20cat	*cat*	[25]
pLS20rco	*cat rco*_*LS20*_::*kan*	[23]
pUCG18T	*kan* (thermostable)	[16]
pUC19-K7010	*ble*	[33]

**Table 2:** Oligonucleotides used in this study

Oligonucleotides	Sequence (5′ → 3′)
inverse-F	CATGATTACGAATTCGAGC
inverse-R	GTCATAGCTGTTTCCTGTGTG
ori-F	GGAAACAGCTATGACCATATGTTCCTTAAGGAACGTACAG
kan-F	GGAAACAGCTATGACTCGACCGAAAAATAAATATAAATC
kan-R	GAATTCGTAATCATGCATATGTCAAAATGGTATGCG
epr-uF	CCGGAATCGGCAAGCTCG
epr-uR	GCTCAGTTAATTCTTTGATGCCATGTGCCGTCTGACAGCACTTTG
epr-dF	GCAATCGCCCTAATATATGGAAGACGGCACAGCAATCCG
epr-dR	CGGCTTGTTCATCGTATCAAATG
rpsO-F	ATGGCATCAAAGAATTAACTGAGC
rpsO-R	CCAAATCATATTTAGCCCCAGTTACC
dam-F	CTAAATATGATTTGGAGGTGAAACAGGATGATGAAGAAAAATCGCGCTTTTTTGAAG
dam-R	CCGAATAGCAAAAAACTGGCTGTTTCATCCGCTTCTCCTTGAG
phleo-F	CCAGTTTTTTGCTATTCGG
phleo-R	CATATATTAGGGCGATTGC

### Construction of *B. subtilis* strains

*Bacillus subtilis* was transformed by its natural competence as previously described [32]. Bacterial cells were cultured on LB plates overnight at 37 °C and inoculated into 10-mL MDCH medium containing 2% (w/v) glucose, 3 mM MgSO_4_, 11-mg L^−1^ ferric ammonium citrate, 10.7-g L^−1^ KH_2_PO_4_, 6.0-g L^−1^ K_2_HPO_4_, 1.0-g L^−1^ trisodium citrate, 0.005% l-tryptophan, 0.1% casamino acids, and 0.05% yeast extract. Cells were grown to an optical density of 0.2 at 600 nm (OD_600_) and incubated with shaking at 180 rpm at 37 °C. When OD_600_ reached 1.3–1.5, 10-mL MD medium containing 2% glucose, 3 mM MgSO_4_, 11-mg L^−1^ ferric ammonium citrate, 10.7-g L^−1^ KH_2_PO_4_, 6.0-g L^−1^ K_2_HPO_4_, and 1.0-g L^−1^ trisodium citrate was added, and cells were incubated further at 37 °C for 1 h. Subsequently, 1-mL the culture was taken to a new tube and incubated with 100–1000-ng DNA (PCR fragment, extracted genome, or plasmid) at 37 °C for 1 h. Next, cells were plated on LB medium supplemented with appropriate antibiotics and incubated at 37 °C overnight.

To construct strain TSU077, the *dam* methylase gene of *E. coli* was inserted into the *epr* locus of *B. subtilis* 168 as follows. Upstream and downstream regions of the *epr* gene were amplified from the *B. subtilis* 168 chromosome by PCR using primer pairs of epr-uF/epr-uR and epr-dF/epr-dR (Table 2), respectively. The promoter region of *rpsO* was amplified from the *B. subtilis* 168 chromosome using the rpsO-F/rpsO-R primer pair (Table 2). The coding region of *dam* was amplified from the DNA of *E. coli* DH5α using the dam-F/dam-R primer pair (Table 2). The phleomycin resistance gene (*ble*) was amplified from pUC19-K7010 [33] using the phleo-F/phleo-R primer pair (Table 2). The five PCR fragments were designed to be connected by recombinant PCR, which resulted in a single DNA fragment with the following configuration: (i) upstream *epr* region, (ii) *rpsO* promoter, (iii) *dam* gene, (iv) phleomycin resistance gene *ble*, and (v) downstream *epr* region. This DNA fragment was used to transform *B. subtilis* 168. One of the randomly selected phleomycin resistant transformants, whose correct construction was confirmed to contain the *dam* gene under the control of *rpsO* promoter at the *epr* locus, was named strain TSU077. Plasmid pLS20cat was introduced into TSU077 by conjugation from PKS11 [22] to yield strain STM1.

### Construction of plasmids

**Fig. 2:**
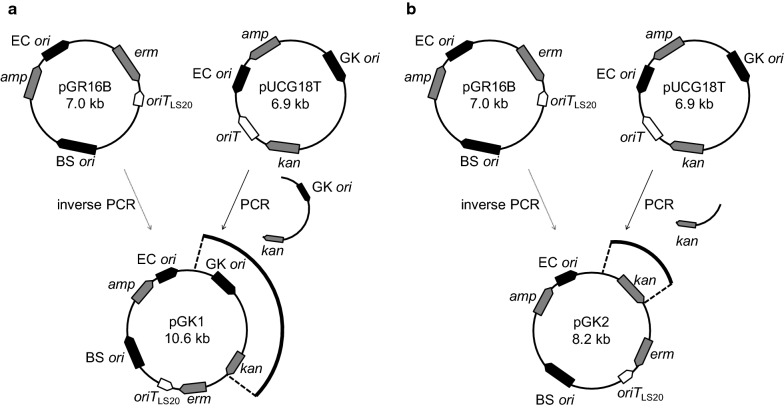
Construction of plasmids pGK1 and pGK2. **a** The *E. coli*–*B. subtilis* shuttle vector pGR16B contains the ampicillin resistance gene (*amp*), the replication origin containing the Rep gene of rolling circle plasmid pTA1015 (BS *ori*), the replication origin of pUC19 (EC *ori*), the erythromycin resistance gene (*erm*), and *oriT*_LS20_. pGK1 was constructed from pGR16B by inserting the segment containing both the functional *Geobacillus* replication origin (GK *ori*) and the thermostable kanamycin resistance gene (*kan*) of pUCG18T. **b** pGK2 was also constructed from pGR16B by inserting only the segment containing *kan* of pUCG18T

The mobilizable plasmid pGK1 was constructed as follows (Fig. 2). pGR16B is a mobilizable plasmid containing *oriT*_LS20_ constructed previously (Table 1) [24]. A PCR fragment corresponding to linearized pGR16B was amplified from circular plasmid DNA of pGR16B as template with the specific primer pair of inverse-F/inverse-R (Table 2). Another fragment containing the replication origin region and thermostable kanamycin resistance gene was amplified from plasmid pUCG18T (Table 1) using the ori-F/kan-R primer pair (Table 2). The two fragments were connected and circularized using NEBuilder HiFi DNA Assembly Master Mix (New England Biolabs) to obtain pGK1. Plasmid pGK2 was constructed in a similar way as pGK1, except that the latter fragment was designed to contain only the replication origin of pUCG18T using the kan-F/kan-R primer pair (Table 2).

### Conjugation and mobilization

Unless indicated otherwise, conjugation and or mobilization experiments were performed by mixing late exponentially growing donor cells (OD_600_ between 0.6 and 1) with recipient cells from a culture whose OD_600_ was less than 2. Thus, donors were cultured overnight in 5 mL of LB liquid medium with appropriate antibiotics with shaking at 180 rpm at 37 °C. Recipients were cultured overnight on LB plate at 65 °C. The donor culture was diluted to OD_600_ 0.05 in 5 ml of fresh LB medium supplemented with 1 mM IPTG and incubated with shaking at 180 rpm at 37 °C. The recipient cells were harvested from a LB plate and suspended in 50 mL of LB medium in a baffle flask to give OD_600_ 0.05. When the donor grew to OD_600_ 0.5–1.0, 1 mL of the donor culture was mixed with 9 mL of recipient culture at OD_600_ less than 2.0. After incubation at 37 °C for 15 min, serially dilutions were plated onto LB agar plates containing appropriate antibiotic(s), and incubated overnight at 37 °C to select the donor cells, and at 65 °C to select either recipients or transformants (transconjugants). Colony forming units (CFU) of total recipients and transconjugants were then determined to calculate mobilization efficiencies per recipient cell using the following formula: CFU of transconjugants/CFU of total recipients × 10^6^ (ppm).

## Results

### Inability of pLS20cat to transfer itself from *B. subtilis* to *G. kaustophilus*

pLS20cat, originally isolated from *B. subtilis* natto, has been known to transfer itself to other *B. subtilis*-related bacteria via conjugation [20]. A conjugation assay between *B. subtilis* donor strain STM1 [(Table 1); *trpC2 epr*::(P*rpsO*-*dam, ble*) pLS20cat] and *G. kaustophilus* recipient strain MK244 (Δ*pyrFR*, Δ*GK1378*–*GK1390*, Δ*GK0343*–*GK0346*) [17] failed to form colonies on chloramphenicol-containing plates after incubation at 65 °C in our repeated experiments (data not shown), indicating that pLS20cat did not transfer itself to *G. kaustophilus*. At least three possible reasons may explain this failure: (i) pLS20cat could not transfer itself to *G. kaustophilus*, (ii) pLS20cat could transfer itself to *G. kaustophilus* but is unable to replicate in *G. kaustophilus*, and/or (iii) pLS20cat could transfer itself to *G. kaustophilus* but the gene for chloramphenicol acetyl transferase (*cat*) did not function in *G. kaustophilus* at 65 °C. Since we obtained positive results in a parallel strategy involving the mobilizable plasmid (see below), we did not investigate further the underlying mechanism why pLS20cat was unable to transfer itself to *G. kaustophilus.*

### Mobilization of pGK1 from *B. subtilis* to *G. kaustophilus* mediated by pLS20cat

**Fig. 3:**
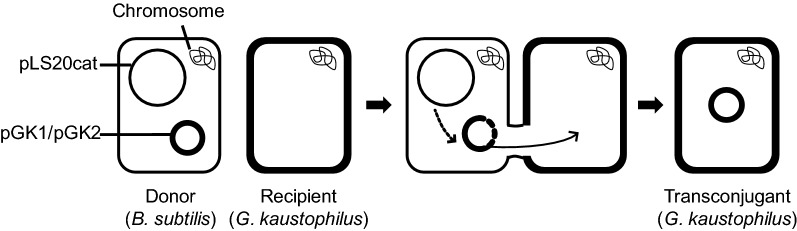
Schematic presentation of the mobilization process described in this study. Large light circle, conjugative plasmid pLS20cat; small bold circle, mobilizable plasmid pGK1 or pGK2; light rounded rectangle, *Bacillus subtilis* donor; bold rounded rectangle, *Geobacillus kaustophilus* recipient

Because pLS20cat is too large to be manipulated in vitro, we instead constructed a smaller mobilizable plasmid, pGK1. pGR16B contains *oriT*_LS20_ and was previously shown to be mobilized with the help of pLS20cat from donor *B. subtilis* to recipient *B. subtilis* [24, 27]. pUCG18T is an *E. coli*-*Geobacillus* shuttle plasmid with *G. stearothermophilus* pBST1 replicon, which shares homology with the family of theta replicons, and a thermostable kanamycin resistance gene [16]. pGR16B was modified by inserting a fragment containing the *Geobacillus* replication origin and the thermostable kanamycin resistance gene of pUCG18T to yield pGK1 (Fig. 2). On the other hand, we constructed the donor strain YNB051, which contains two chromosomal cassettes besides harboring plasmids pLS20cat and pGK1. The first cassette, located at the *epr* locus, contains a copy of the *E. coli dam* gene under the control of the strong and constitutive *rpsO* promoter. As explained in the “Background” section, Dam-mediated methylation in the donor strain protects the DNA against digestion by the *G. kaustophilus* restriction enzyme GKP09 [16]. The second cassette, located at the *amyE* locus, contains a copy of *rap*_*LS20*_ under the control of the IPTG-inducible P*spank* promoter. The pLS20cat gene *rap*_*LS20*_ encodes an anti-repressor that is necessary to activate the conjugation genes of pLS20. Thus, the conjugation genes are by default switched off due to repression of the main conjugation promoter P_*c*_ by the pLS20cat-encoded repressor Rco_LS20_. The anti-repressor Rap_LS20_ is required to activate expression of the conjugation genes [23, 34]. Ectopic expression of *rap*_*LS20*_ is therefore expected to stimulate expression of the pLS20cat conjugation genes and thereby favor mobilization of pGK1. Next, experiments were performed to study possible pLS20cat-mediated mobilization of pGK1 from *B. subtilis* donor strain YNB051 into *G. kaustophilus* MK244 recipient strain (Fig. 3). In short, to mobilize pGK1, aliquots of cultures of the donor strain, grown in the presence of 1 mM IPTG, and the recipient strain were mixed and incubated for 15 min at 37 °C. Next, appropriate dilutions of the mixture were spread onto kanamycin-containing LB agar plates and incubated overnight at 65 °C. *G. kaustophilus* transconjugants were selected after overnight growth at 65 °C on LB plates supplemented with kanamycin. Since *B. subtilis* is unable to grow at 65 °C, the resulting colonies correspond to *G. kaustophilus*. In addition, control experiments in which cells of the *G. kaustophilus* strain MK224 were plated on LB agar plates supplemented with Km did not result in Km-resistant colonies showing that the kanamycin marker used is a reliable marker. Moreover, *G. kaustophilus* and *B. subtilis* can be distinguished by their distinct colony morphologies. The presence of pGK1 in kanamycin-resistant *G. kaustophilus* colonies obtained after overnight growth at 65 °C was unequivocally demonstrated by performing colony PCR on randomly chosen colonies using three different sets of primers. One of these primer sets amplified a DNA region of pGK1, the second and third primer set amplified regions that are specific for *G. kaustophilus* and *B. subtilis*, respectively. PCR fragments of the expected sizes were obtained for the pGK1 and *G. kaustophilus*-specific primer sets. However, no PCR product was obtained using the *B. subtilis* specific primer set, whereas a PCR product of expected size was obtained using this primer set when a colony of *B. subtilis* was used (data not shown). Together, these results demonstrate that the kanamycin-resistant colonies grown at 65 °C correspond to genuine transconjugants.

### Requirements to mobilize pGK1 from *B. subtilis* to *G. kaustophilus*

The mobilization of pGK1 mediated by pLS20cat described above may require all or some of the four factors: (i) the thermostable kanamycin resistance gene, (ii) the *Geobacillus* replication origin on pGK1, (iii) *dam* expression, and (iv) *rap*_LS20_ overexpression in the donor. Because pGK1 possesses an erythromycin resistance gene originated from pGR16B, erythromycin was used as the alternative antibiotic for selection, but no colony appeared on the plates incubated at 65 °C (data not shown). In addition, randomly selected kanamycin-resistant colonies failed to grow on LB agar plates containing erythromycin at 65 °C (data not shown). These results suggested that the erythromycin resistance gene was not functional in *G. kaustophilus* at high temperatures and that the thermostable kanamycin resistance gene on pGK1 was indispensable.

**Fig. 4:**
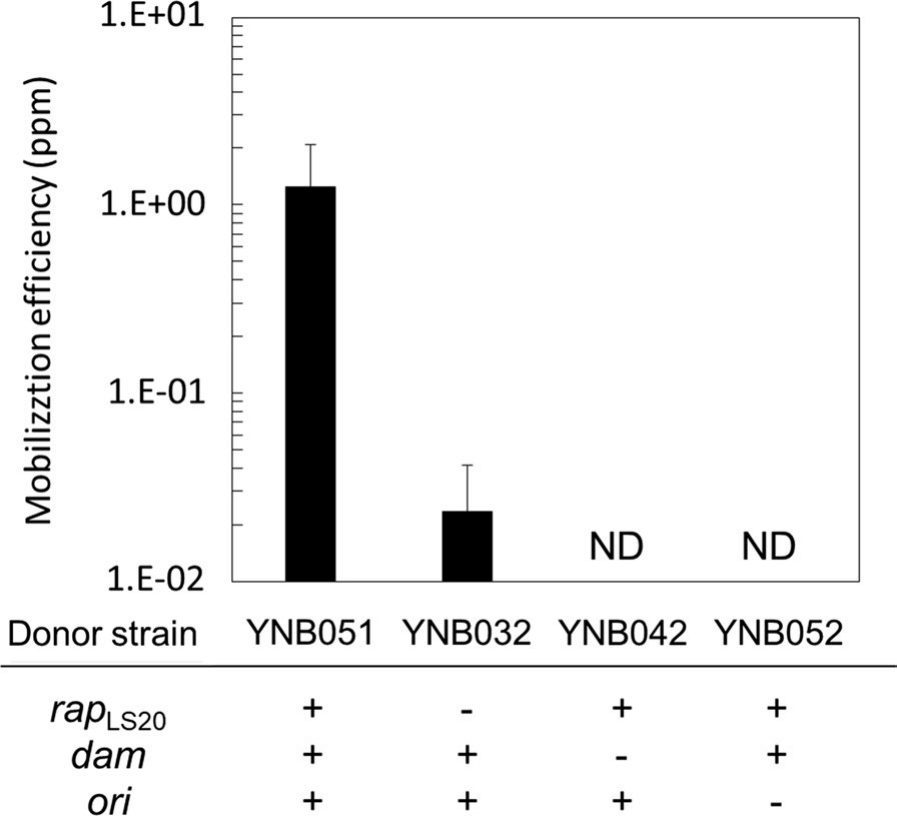
Mobilization of pGK1 and pGK2 with the help of pLS20cat. Liquid cultures of the recipient strain MK244 and one of the donor strains (YNB051, YNB032, YNB042, or YNB052) were mixed for pGK1 or pGK2 mobilization and were plated on LB medium containing kanamycin (K) for transconjugants and no antibiotic for the recipient grown at 65 °C. CFU was determined to calculate mobilization efficiencies. Values are expressed as means with standard deviations from three independent experiments. *ND* not detected (< 1.0 × 10^−2^ ppm)

To test the importance of Dam methylation and ectopic induction of *rap*_*LS20*_, the following two additional donor strains harboring both pLS20cat and pGK1 were constructed. First, strain YNB032, expressing the *dam* gene but lacking the inducible *rap*_*LS20*_ gene. And second, YNB042 containing the inducible *rap*_*LS20*_ gene but lacking *dam*. In addition, to test the effect of the *Geobacillus* replication origin, another derivative of pGR16B was constructed containing the thermostable kanamycin gene but not the replication origin for *Geobacillus* (Fig. 2). This plasmid, named pGK2, was introduced into the *B. subtilis* strain harboring pLS20cat, expressing the *dam* gene, and containing the inducible *rap*_*LS20*_ gene, to yield strain YNB052. When YNB032 (lacking the *rap*_LS20_ cassette) was used as donor, pGK1 mobilization was approximately 50-fold less efficient than when strain YNB051 was used (Fig. 4). This demonstrated that ectopic expression of *rap*_*LS20*_ was not essential for pGK1 mobilization but significantly increased the pGK1 mobilization efficiency. Contrary to our expectations, a stimulatory effect on pGK1 mobilization frequency was not observed when a pLS20cat derivative lacking a functional *rco*_LS20_ was used (see “Discussion”). More dramatic outcomes were obtained for the other two factors studied: no transconjugant appeared when strains YNB042 (lacking *dam*) or YNB052 (harboring pGK2 instead of pGK1) were used as a donor. These results suggested that Dam-methylation of the DNA as well as a replication origin that is functional in *Geobacillus* were essential for successful pLS20cat-mediated mobilization of the mobilizable plasmid into *G. kaustophilus*.

### The type I RM systems of *G. kaustophilus* did not affect pLS20cat-mediated mobilization of pGK1

**Fig. 5:**
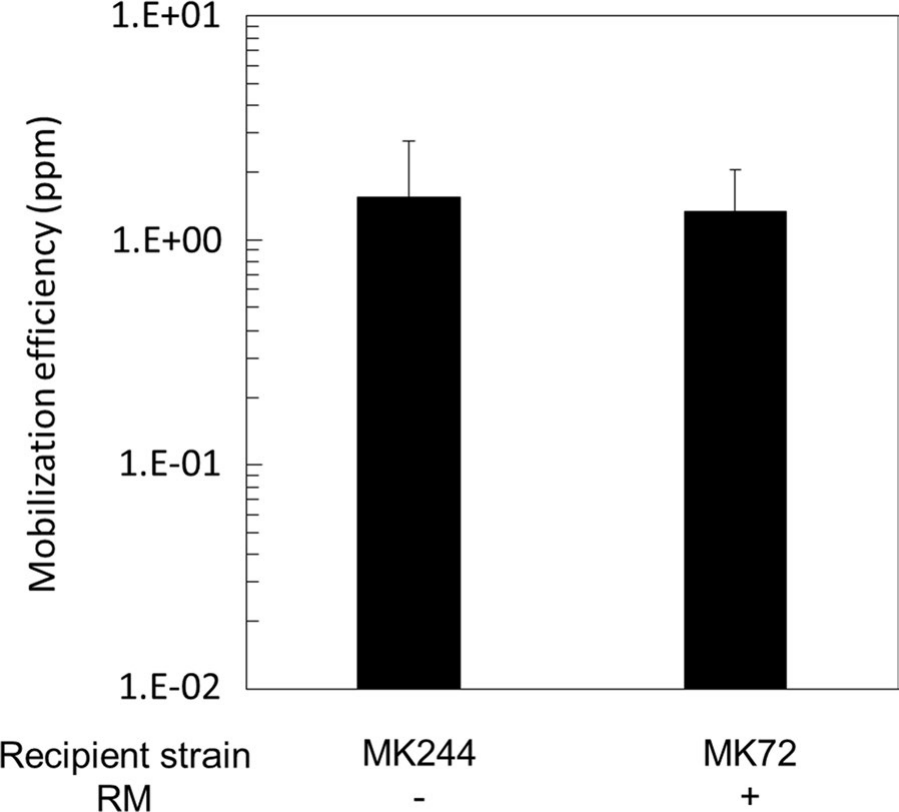
pGK1 mobilization with or without two sets of type I RM system in *G. kaustophilus*. Liquid cultures of the recipient strain (MK244 or MK72) and the donor strain YNB051 were mixed for pGK1 mobilization and plated on LB medium containing kanamycin (K) for transconjugants and no antibiotic for the recipient grown at 65 °C. CFU was determined to calculate mobilization efficiencies. Values are expressed as means with standard deviations from three independent experiments

A major function of the RM systems is to digest and thereby inactivate incoming foreign DNAs that are not properly methylated. Thereby, RM systems are a first-line defense mechanism of most bacteria to protect them against invading DNAs including bacteriophages and (conjugative) plasmids [35]. *G. kaustophilus* HTA426 possesses two type I RM systems: *GK0343* (M subunit)-*GK0344* (S subunit)-*GK0346* (R subunit) and *GK1380* (M subunit)-*GK1381* (S subunit)-*GK1382* (R subunit). In addition, it possesses a type II RM system, namely *GKP09* (endonuclease)-*GKP08* (methylase), which is responsible for a methylation pattern similar to that of *E. coli dam* (5′-G^*N*6m^ATC-3′). As described above, *dam* expression in the donor *B. subtilis* was necessary for pGK1 mobilization to *G. kaustophilus*, indicating that pGK1 DNA has to be properly methylated by Dam to be protected from digestion by the type II restriction enzyme GKP09. *G. kaustophilus* strain MK244 used in the mobilization experiments described above was deleted for the two type I RM systems (Δ*GK1378*–*GK1390* Δ*GK0343*–*GK0346*) [17]. To analyze the effect of the type I RM systems we compared the pGK1 mobilization efficiencies from donor strain YNB051 into the two isogenic recipient strains lacking and containing the two type I RM systems (strains MK244 and MK72, respectively). Figure 5 shows that similar pGK1 mobilization efficiencies were obtained with the two recipient strains, indicating that the two type I RM systems did not affect pGK1 mobilization to a large extent.

### Effects of growth phase of the donor and recipient on mobilization of pGK1

**Fig. 6:**
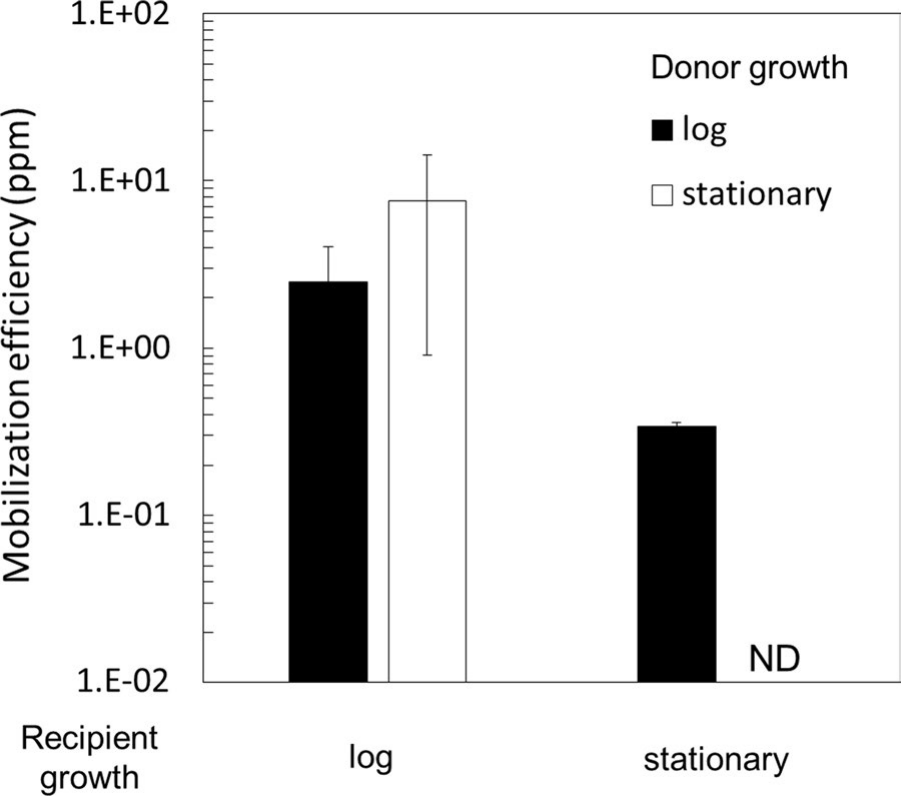
pGK1 mobilization depending on the growth phase of the donor and recipient. Liquid cultures of the recipient MK244 and the donor YNB051 in different growth phases were mixed for pGK1 mobilization and plated on LB medium containing kanamycin (K) for transconjugants and no antibiotic for the recipient grown at 65 °C. CFU was determined to calculate mobilization efficiencies. Values are expressed as means with standard deviations from three independent experiments. *ND* not detected (< 1.0 × 10^−2^ ppm)

In *B. subtilis*, the conjugation efficiency of pLS20cat is affected by the growth phase of the donor, not the recipient [23]. We examined therefore whether the growth phase of either the donor or recipient strain affected mobilization efficiencies of pGK1. Thus, pGK1 mobilization assays were conducted using donor YNB051 and recipient MK244 in different growth phases (Fig. 6). Similar pGK1 mobilization efficiencies were obtained when either exponentially or stationary growing donor cells were used for mating with exponentially growing recipient cells (Fig. 6). This suggests that ectopic expression of *rap*_LS20_ in the YNB051 donor overrules the native quorum-sensing regulatory mechanism responsible for repression of the conjugation genes during the stationary phase. Interestingly, about tenfold lower pGK1 mobilization efficiencies were obtained when recipient cells harvested at stationary phase were mated with exponentially growing donor cells. The affect was even more pronounced when stationary phase recipient cells were mated with stationary phase donor cells. In this latter set up, the mobilization efficiencies dropped more than 100-fold compared to similar mating using exponentially instead of stationary phase recipient cells. These results show that the growth phase of the recipient cells cardinally affects the efficiency of pGK1 mobilization, and that maximum pGK1 mobilization efficiencies are obtained in exponentially growing recipient cells.

### Effect of *oriT*_LS20_ function in pLS20cat on mobilization of pGK1

**Fig. 7:**
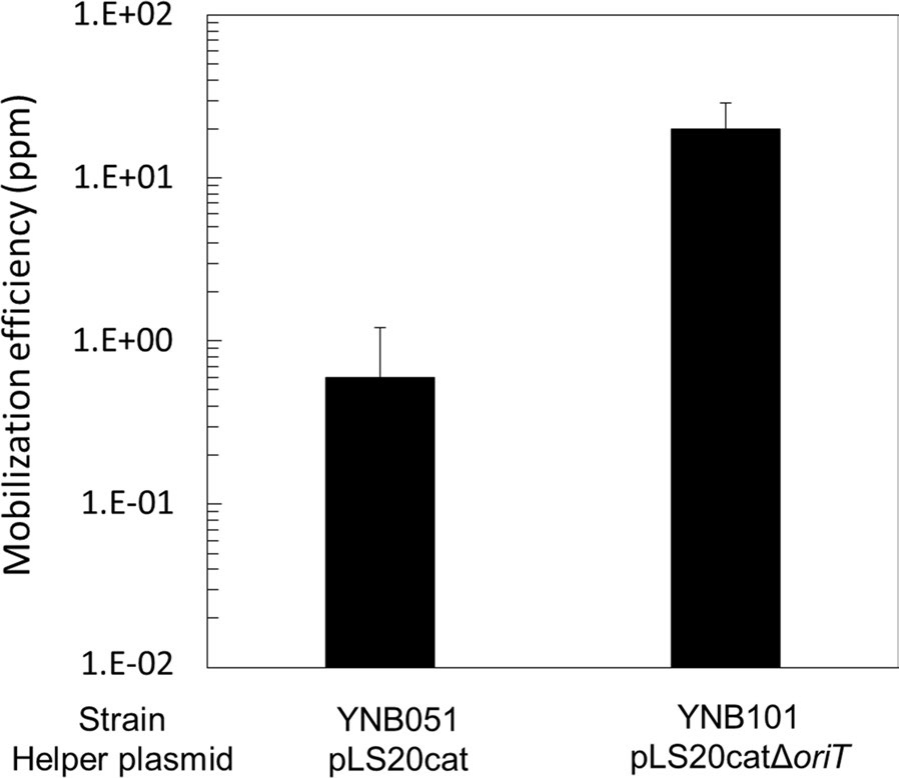
pGK1 mobilization with the help of pLS20cat or pLS20catΔ*oriT*. Liquid cultures of the recipient strain MK244 and one of the donor strains (YNB051 or YNB101) were mixed for pGK1 mobilization and plated on LB medium containing kanamycin (K) for transconjugants and no antibiotic for the recipient grown at 65 °C. CFU was determined to calculate mobilization efficiencies. Values are expressed as means with standard deviations from three independent experiments

Conjugation of pLS20cat occurs with a higher frequency than mobilization of co-resident elements [29]. In the case of heterologous relaxosome complexes, this may be explained by lower affinity/compatibility between the relaxosome complex, and/or affinity to the T4 coupling protein that is believed to recruit the relaxosome complex to the pore. In the case of homologous relaxosome complexes (i.e. a mobilizable and conjugative plasmid containing the same *oriT*) it may be that the *oriT* of the conjugative plasmid is recognized with higher efficiency by the relaxosome complex for instance due to different local conformation of the DNA near the *oriT*. In any case, whatever the underlying reason, co-residence of a mobilizable element containing the same *oriT* as present on the conjugative element is expected to result in competition for binding the limited amount of relaxosome proteins. In other words, the presence of a pLS20cat derivative lacking its *oriT*, may favor recruitment of the relaxosome proteins to the *oriT* of pGK1. Previously, we have constructed a derivative of pLS20cat lacking its *oriT*. As expected, this derivative, named pLS20catΔ*oriT*, has lost its conjugative self-transfer ability [29]. To test the hypothesis mentioned above we compared pGK1 mobilization efficiencies to *G. kaustophilus* MK244 mediated by pLS20cat or pLS20catΔ*oriT*. The results presented in Fig. 7 show that pGK1 was mobilized about 30-fold more efficiently by pLS20catΔ*oriT* than pLS20cat.

## Discussion

Here, we present a novel, rapid and versatile technique in which a plasmid is mobilized from *B. subtilis* donor to a *G. kaustophilus* recipient mediated by the conjugative plasmid pLS20cat. Although successful transfer of pLS20cat to *G. kaustophilus* was not obtained, we showed that it could mobilize pGK1. pGK1 has an erythromycin and a kanamycin resistance gene. However, *G. kaustophilus* transconjugants were only obtained when the mating mixtures were selected on kanamycin. The erythromycin resistance gene was derived from pE194 [36] isolated from mesophilic *Staphylococcus aureus* [37], suggesting that the erythromycin resistance gene does not function at 65 °C or in *G. kaustophilus*.

Plasmid replication can be divided into two types: rolling circle replication and theta replication [38]. In theta replication, a replication initiator (Rep) protein recognizes the replication origin of a plasmid and facilitates the melting of two strands. This step is followed by the recruitment of host factors to the replication origin and initiation and elongation of DNA. Most theta replication plasmids use a plasmid-encoded Rep, but some require a host-encoded Rep to initiate replication [39]. pLS20cat is classified as a theta replication plasmid [21]. The minimal region needed for its replication in *B. subtilis* identified previously did not contain any open reading frame, suggesting that theta replication of pLS20cat depends on the host machinery. Despite several attempts, we were unable to obtain successful conjugative transfer of pLS20cat from *B. subtilis* to *G. kaustophilus*. The apparent inability of pLS20cat to transfer itself to *G. kaustophilus* may be due to several reasons. However, since pGK1 is successfully mobilized, the failure of pLS20cat transfer is most likely neither due to the inability of pLS20cat-harboring *B. subtilis* donor cells forming stable mating pairs with *G. kaustophilus* nor to the inability of forming a functional connecting pore between the heterologous donor and recipient cells. We are currently investigating different possibilities that may cause the lack of successful pLS20cat transfer.

In this study, we have demonstrated that pLS20cat-mediated mobilization is a useful strategy to modify *G. kaustophilus*. In addition, by testing various conditions we have achieved to improve the mobilization frequency of our test plasmid pGK1 to transform up to 20 ppm recipient cells. The ectopic overexpression of *rap*_LS20_ in the donor *B. subtilis* turned out to significantly improve the mobilization because under those conditions we found that the mobilization efficiency was about 50-fold higher (Fig. 4). We previously constructed a donor strain harboring *rco*_LS20_-deficient mutant of pLS20cat, called pLS20rco [23]. Rco_LS20_ is the master repressor of the conjugation genes, and hence all conjugation genes are constitutively expressed in pLS20rco, which probably poses a burden to the plasmid and/or the donor cells [23]. To avoid this, a copy of *rco*_LS20_ was placed onto the chromosome of strain PKS86 under the control of the IPTG-inducible P*spank* promoter [23]. To examine pGK1 mobilization mediated by pLS20rco, the *E. coli dam* gene and pGK1 were introduced into PKS86 (harboring pLS20rco), which was maintained in the presence of 1 mM IPTG but mated with the recipient *G. kaustophilus* without IPTG. In our repeated experiments, this donor exhibited no difference in pGK1 mobilization to *G. kaustophilus* compared with the donor YNB032 (data not shown). Thus, whereas pGK1 mobilization frequencies increased when the expression levels of the pLS20cat conjugation genes were augmented by overexpressing *rap*_*LS20*_, the frequencies were not elevated in the absence of *rco*, which also causes an increase in expression of the conjugation genes. At present we do not have an explanation for these latter results.

In the previous studies on conjugative plasmid transfer from *E. coli* to *G. kaustophilus*, *dam*-methylated plasmids exhibited more efficient mobilization than un-methylated ones, and inactivation of the two type I RM systems also increased the mobilization efficiencies [16, 17]. In this study, we conducted two experiments. Firstly, the two donor strains YNB051 (*dam*^+^) and YNB042 (*dam*^−^) were compared (Fig. 4) to show that *dam* methylation was necessary to mobilize pGK1 (Fig. 4). Secondly, the two recipient strains MK244 (type I RM^−^) and MK72 (type I RM^+^) were compared mating with the donor YNB051 to demonstrate that inactivation of the two type I RM systems did not have major effects on pGK1 mobilization (Fig. 5). Although possible presence of putative type I RM systems in *B. subtilis* could provide a logical explanation for these results, it is quite difficult to imagine that these putative type I RM systems would have the same recognition sites as the two type I RM systems present in *G. kaustophilus*. Alternatively, pGK1 might not have the sequences recognized by the two type I RM systems, which have not been defined yet. At this moment, therefore, we are unable to explain properly why the two type I RM systems did not affect pGK1 mobilization from *B. subtilis* to *G. kaustophilus*. The results suggested that there could be unknown differences in the set ups between *E. coli* versus *B. subtilis* conjugation with *G. kaustophilus.*

Previous studies showed that the Phr*LS20 quorum-sensing peptide is the determining factor to activate the conjugation process from *B. subtilis* donors to *B. subtilis* recipients by regulating de-repression of the of the P*c* promoter for conjugation genes [23]. In standard conjugation experiments, the concentration of the Phr*LS20 peptide secreted by donor cells was distributed evenly and continuously in the culture due to shaking. Consequently, Phr*LS20 concentrations are low during exponential growth and start to increase rapidly when the culture reaches stationary growth. The high Phr*LS20 levels indeed cause a rapid inhibition in conjugation efficiencies when donor cells reach the stationary phase growth. However, this scenario does not explain the very low conjugation levels observed during the start of the experiment and why they built up to maximum levels near the end of the exponential growth phase. This growth-dependent increase in conjugation efficiencies during early to late exponential growth phase are most probably due to the particular experimental set up in which the starting donor culture corresponds to a culture being inoculated with very late stationary grown donor cells (overnight grown culture). Phr*LS20 levels will have accumulated to very high levels in the medium and inside the cells, which caused inactivation of RapLS20 until the intracellular levels of Phr*LS20 have diminished by, for instance, rapid cell growth. This view was supported was by the fact that high levels of conjugation were obtained throughout the exponential growth phase when the experiment was started using a donor culture that was inoculated with late exponentially growing cells instead of very late stationary cells. In these series of experiments, possible effects on the growth phase of the recipient cells were also tested. No indications were found that the growth phase of the recipient cells had a large effect on pLS20cat conjugation efficiency [23]. Here, we investigated possible growth phase effects on pGK1 mobilization from the *rap*_LS20_-overexpressing *B. subtilis* donor to the *G. kaustophilus* recipient (Fig. 6). When exponentially growing recipient cells were used, similar mobilization efficiencies were obtained for exponentially and stationary phase donor cells, indicating that the ectopic expression of *rap*_LS20_ overruled the quorum-sensing function of Phr*_LS20_ in the donor cells during the stationary phase as was also observed before [23]. However, whereas the growth phase of the recipient cells did not affect much the conjugation efficiency of pLS20cat, it did largely influence the mobilization efficiency of pGK1. The effects were especially pronounced in crosses using donor and recipient cells that were both in their stationary phase. It has to be taken into account that whereas the results obtained for pLS20cat conjugation concerned a homologous system (i.e. plasmid transfer between the same bacterial species), those obtained for pGK1 mobilization concerned a heterologous system (i.e. transfer from *B. subtilis* to *G. kaustophilus*). The initial step for conjugation/mobilization includes the formation of a mating pair by which the donor cell recognizes and establishes contact with the recipient cell, most probably by surface-located proteins present on the donor and recipient cell. The proteins involved in mating pair formation are unknown for the pLS20 system. Possibly, the observed growth-phase dependent effects on mobilization efficiency are because the *G. kaustophilus* receptor for mating pair formation is different from that encoded by *B. subtilis* and may be recognized less efficiently by the pLS20cat system. In addition, or alternatively, it may be that this receptor is not or less expressed during stationary phase in *G. kaustophilus* than in *B. subtilis*. In the same line of reasoning, it may be that stationary phase *G. kaustophilus* cells have an altered cell surface structure causing that the receptor is not properly exposed for recognition by the pLS20cat system. Moreover, we cannot exclude that the lower mobilization efficiencies observed during stationary phase are due to other or additional reasons. In any case, whatever the underlying mechanism and how intriguing this may be, we show here that optimum mobilization efficiencies are obtained using exponentially growing recipient cells.

We also found that the use of a pLS20cat derivative lacking its *oriT*, pLS20catΔ*oriT*, led to increased pGK1 mobilization efficiencies (Fig. 7). Our preliminary data suggest that, compared with pLS20cat, the use of pLS20catΔ*oriT* also increases significantly (~ tenfold) the mobilization efficiency of large *B. subtilis* chromosomal regions (our unpublished results) [31], indicating that the enhanced mobilization efficiency is a general feature of pLS20catΔ*oriT.* One possible explanation is that the absence of *oriT*_LS20_ on pLS20cat enhances formation of the relaxosome complex at the *oriT*_LS20_ present on co-resident mobilizable element. Similarly, the co-resident mobilizable element containing *oriT*_LS20_ will not experience competition from pLS20catΔ*oriT* to dock onto the cytoplasmic site of the connecting pore.

We have established the method to mobilize the plasmid pGK1 from *B. subtilis* donor into *G. kaustophilus* recipient, and identified various conditions that affect the mobilization efficiency. The optimal conditions to obtain maximum mobilization efficiencies are listed as follows: for the recipients, exponentially growing cells should be used; and for donors, cells should ectopically express both *rap*_LS20_ for the anti-repressor and the *E. coli dam* gene and in addition carry the helper plasmid pLS20catΔ*oriT*. We need to define the best combination of the conditions, but at least we were able to make up to 20 ppm of recipient cells transformed (Fig. 7). To develop further the transformation method of *G. kaustophilus*, the limited number of selection markers available for *G. kaustophilus* that are stable at high temperatures may be an issue. At this moment, three antibiotics in addition to kanamycin, including chloramphenicol, spectinomycin, and thiostrepton, could be usable at 60 °C. However, respective resistance genes remain to be tested [32, 33, 40]. Nevertheless, the transformation method demonstrated in the current study has at least two advantages. First, plasmid mobilization is achieved within 15 min in liquid media, unlike the other conventional systems, which usually require more than 5-h incubation on solid media [41–44]. Second, various *B. subtilis*-related Gram-positive bacteria, such as *B. cereus*, *B. licheniformis*, *B. megaterium*, *B. pumilus*, and *B. thuringiensis*, are susceptible to plasmid transfer mediated by pLS20cat, suggesting that other species in addition to *G. kaustophilus* could be transformed using a method similar to the one implemented in this study.

## Conclusions

We describe a novel method for transforming *G. kaustophilus* using pLS20cat-mediated plasmid mobilization. The donor was modified to acquire DNA methylation mimicking the recipient, and the mobilizable plasmid was equipped with the additional replication origin and thermostable selection marker functioning in the recipient. In addition, ectopic expression of the anti-repressor of the conjugation genes *rap*_LS20_, exponentially growing recipient cells, and elimination of *oriT*_LS20_ from pLS20cat elevated the mobilization frequencies. This system is rapid and easy and enables pGK1 mobilization by simply mixing the donor and recipient in liquid media for only 15 min. A similar concept may be applied to genetically manipulate other Gram-positive thermophilic bacteria that are reluctant to modification by standard techniques.

## Abbreviations

*cat*: chloramphenicol acetyl transferase
CFU: colony forming units
HGT: horizontal gene transfer
LB: lysogeny broth
OD_600_: optical density at 600 nm
*oriT*: origin of transfer
*oriT*_LS20_: *oriT* sequence of pLS20cat
Rep: replication initiator
RM: restriction-modification
